# Characterizing the *Aedes aegypti* Population in a Vietnamese Village in Preparation for a *Wolbachia*-Based Mosquito Control Strategy to Eliminate Dengue

**DOI:** 10.1371/journal.pntd.0000552

**Published:** 2009-11-24

**Authors:** Jason A. L. Jeffery, Nguyen Thi Yen, Vu Sinh Nam, Le Trung Nghia, Ary A. Hoffmann, Brian H. Kay, Peter A. Ryan

**Affiliations:** 1 Queensland Institute of Medical Research and Australian Centre for International and Tropical Health, Brisbane, Australia; 2 National Institute of Hygiene and Epidemiology, Hanoi, Vietnam; 3 Administration of Preventive Medicine and Environmental Health, Ministry of Health, Hanoi, Vietnam; 4 Institute Pasteur, Nha Trang, Vietnam; 5 Centre for Environmental Stress and Adaptation Research, Department of Genetics, University of Melbourne, Victoria, Australia; Duke University-National University of Singapore, Singapore

## Abstract

**Background:**

A life-shortening strain of the obligate intracellular bacteria *Wolbachia*, called *w*MelPop, is seen as a promising new tool for the control of *Aedes aegypti*. However, developing a vector control strategy based on the release of mosquitoes transinfected with *w*MelPop requires detailed knowledge of the demographics of the target population.

**Methodology/Principal Findings:**

In Tri Nguyen village (611 households) on Hon Mieu Island in central Vietnam, we conducted nine quantitative entomologic surveys over 14 months to determine if *Ae. aegypti* populations were spatially and temporally homogenous, and to estimate population size. There was no obvious relationship between mosquito (larval, pupal or adult) abundance and temperature and rainfall, and no area of the village supported consistently high numbers of mosquitoes. In almost all surveys, key premises produced high numbers of *Ae. aegypti*. However, these premises were not consistent between surveys. For an intervention based on a single release of *w*MelPop-infected *Ae. aegypti*, release ratios of infected to uninfected adult mosquitoes of all age classes are estimated to be 1.8–6.7∶1 for gravid females (and similarly aged males) or teneral adults, respectively. We calculated that adult female mosquito abundance in Tri Nguyen village could range from 1.1 to 43.3 individuals of all age classes per house. Thus, an intervention could require the release of 2–78 *w*MelPop-infected gravid females and similarly aged males per house, or 7–290 infected teneral female and male mosquitoes per house.

**Conclusions/Significance:**

Given the variability we encountered, this study highlights the importance of multiple entomologic surveys when evaluating the spatial structure of a vector population or estimating population size. If a single release of *w*MelPop-infected *Ae. aegypti* were to occur when wild *Ae. aegypti* abundance was at its maximum, a preintervention control program would be necessary to ensure that there was no net increase in mosquito numbers. However, because of the short-term temporal heterogeneity, the inconsistent spatial structure and the impact of transient key premises that we observed, the feasibility of multiple releases of smaller numbers of mosquitoes also needs to be considered. In either case, fewer *w*MelPop-infected mosquitoes would then need to be released, which will likely be more acceptable to householders.

## Author Summary

Prevention of dengue disease relies predominantly on controlling the mosquito *Aedes aegypti*. A new control strategy is being developed based on the use of a life-shortening strain of the intracellular bacteria *Wolbachia*, which has been microinjected into *Ae. aegypti* in the laboratory. This strategy aims to eliminate the older and epidemiologically significant individuals from *Ae. aegypti* populations and thus eliminate dengue transmission. However, designing an intervention strategy involving the release of *Wolbachia*-infected mosquitoes requires detailed knowledge of the population dynamics of *Ae. aegypti* at potential release sites. We can then calculate how many mosquitoes need to be released for *Wolbachia* to spread through the wild population. We examined the abundance and distribution of the *Ae. aegypti* population over a 14 month period in a Vietnamese village identified by the Vietnam government as a potential pilot release site. We found considerable spatial variability because the houses that produced high numbers of mosquitoes were different in each survey. We estimated that the adult *Ae. aegypti* female population could be as high as 43 individuals per house. This suggests suppression of the wild *Ae. aegypti* population prior to a release will be necessary, and multiple releases will be required to counteract unpredictable spatial heterogeneity.

## Introduction

Dengue fever (DF) and dengue haemorrhagic fever (DHF) are caused by four antigenically related, but distinct, viruses in the genus *Flavivirus*, family Flaviviridae [1],[2]. Globally, there are an estimated 50 to 100 million cases of DF and several hundred thousand cases of DHF per year, with case-fatality rates for DHF of approximately 5% [3]. As there is no vaccine for DF/DHF, mosquito control is currently the best method for epidemic prevention and control. In most urban centres in the tropics, *Aedes aegypti* (L.) is the most important vector [3].

The obligate intracellular bacteria *Wolbachia* are a promising vector control tool because of the range of alterations that they can have on the life history and reproduction of hosts [4]. Through cytoplasmic incompatibility, which partially or completely sterilizes matings between infected males and uninfected females, *Wolbachia-*infected females have a reproductive advantage over uninfected females. As *Wolbachia* is transmitted maternally, this allows *Wolbachia* to sweep through a population, often to fixation [4]–[6], even if there is a fitness cost associated with carrying *Wolbachia*. A pathogenic strain of *Wolbachia*, termed *w*MelPop, was observed to almost halve the lifespan of adult *Drosophila melanogaster* through over-replication in the central nervous system [7]. This generated interest in the use of *w*MelPop to reduce pathogen transmission by increasing vector mortality [8],[9]. Recently, a stable *w*MelPop infection was established in *Ae. aegypti* through embryonic microinjection, resulting in a life-shortening phenotype [10].

To use *Wolbachia* in an applied manner for controlling mosquito vectors, one of the critical parameters to understand is the unstable equilibrium, or the number of *Wolbachia*-infected individuals that must be released for infection to spread and become established in a wild population. This will determine whether a release is logistically and economically feasible [11]. Thus, it is important to have a good understanding of the target insects' potential abundance at the nominated release site [12], since success in introducing modified mosquitoes will be greater when wild mosquito population densities are low [6]. However, estimates of total adult population numbers of wild insects are difficult to obtain. For *Ae. aegypti*, these estimates have often been made by extrapolating from the standing crop of pupae [13]–[15], which ideally need to be based on large, replicate surveys [16]. Spatial heterogeneity and seasonal fluctuations in the abundance of the target insect are also important to understand [12]. Such data can then be used to inform models, ideally those with spatially explicit components [17], to predict the spread of *Wolbachia* into the native mosquito population.

The location of our study was Tri Nguyen village, on Hon Mieu island, central Vietnam. Because of its small size (611 houses) and physical separation from the mainland, this village is being considered by the Government of Vietnam as a potential pilot site for a *Wolbachia*-based intervention against *Ae. aegypti*. Our aims were to 1) use contemporary quantitative sampling tools to define the temporal and spatial patterns of *Ae. aegypti* abundance; 2) use household-level estimates of pupal abundance to derive daily estimates of adult mosquito emergence, and extrapolate this to village-wide estimates of total adult abundance; and 3) use these estimates of adult mosquito abundance, in conjunction with contemporary estimates of the unstable equilibrium, to calculate the number of *Wolbachia*-infected individuals that would need to be released. Addressing these aims is a critical first step for planning a pilot population replacement strategy involving the release of modified mosquitoes in Tri Nguyen village. This is the first time that modelled release ratios have been combined with population abundance estimates from a potential pilot release site to give a realistic indication of the number of mosquitoes that would need to be released for *Wolbachia* to drive through a wild *Ae. aegypti* population.

## Materials and Methods

### Study site

**Figure 1:**
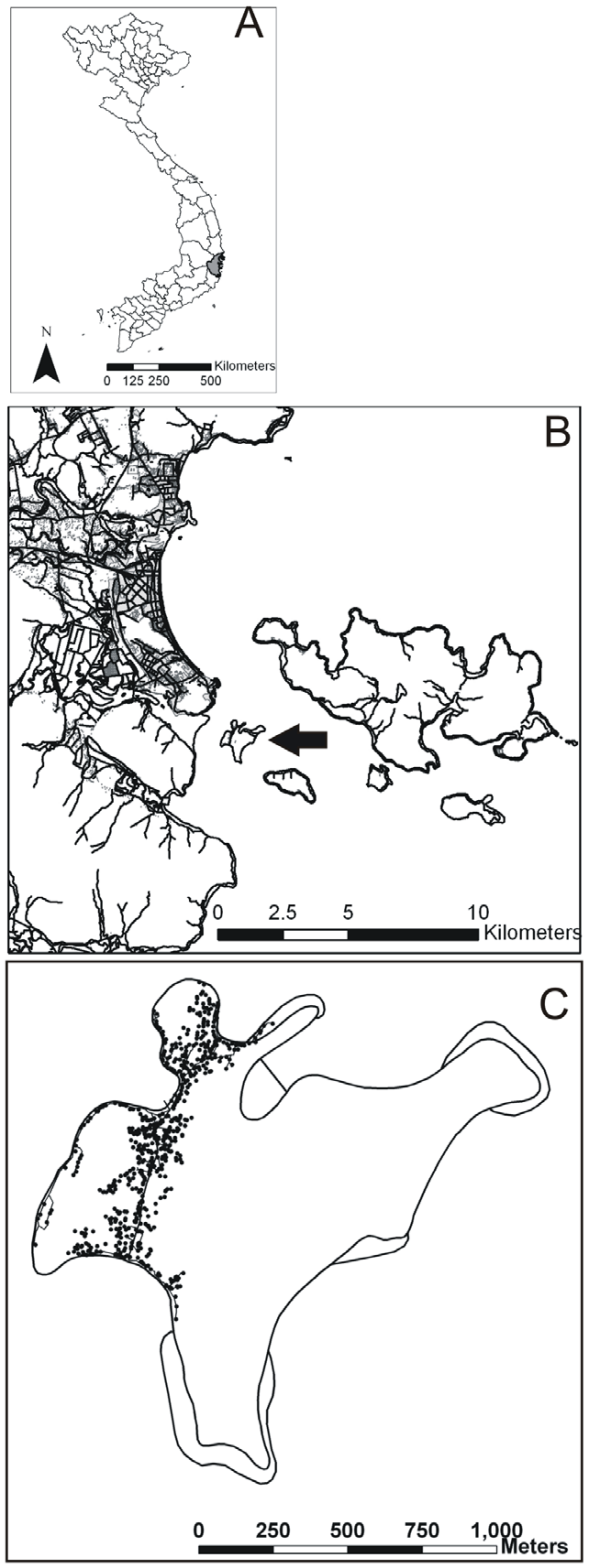
Map showing the location of the study site. A) Khanh Hoa province, central Vietnam. B) Nha Trang city, Khanh Hoa province. Hon Mieu island is marked with an arrow. C) Hon Mieu island, and the 611 houses that make up Tri Nguyen village.

Tri Nguyen village is located on Hon Mieu island (12°11′26″N, 109°13′31″E), Khanh Hoa province, central Vietnam (Fig. 1). The island lies approximately 1 kilometer from Nha Trang city on the mainland, and is approximately 1.2 km^2^ (117 ha) whilst the village is approximately 0.2 km^2^ (22 ha) in size. The houses in the village are located in a rough north-south pattern on the western side of the island. There is no piped water on the island and householders store their water in containers. These water containers are the major development sites for *Ae. aegypti* immatures in the village. Dengue transmission occurs sporadically in the village, probably as a result of introductions of virus via viremic individuals from the nearby mainland. Between 2003 and January 2007 there were 22 cases of DHF recorded in the village (Ministry of Health, unpublished data).

To define the standing crops of III/IV instars and pupae, containers were classified into 10 categories: molded tanks (∼2000 L), cylindrical tanks (1500–2000 L), box tanks>500 L, box tanks<500 L, standard jars (>100 L), small jars (<100 L), ant traps, aquariums, flower vases, and other discards. To fill these containers, householders rely on water collected from their roofs, from five public wells (which are slightly brackish), or from water vendors who transfer water from the mainland on boats.

As there were no street addresses, all houses were geo-referenced with handheld GPS60 units (Garmin Ltd., Olathe, Kansas) using the Universal Transverse Mercator coordinate system and WGS-84 datum. Coordinates were taken at the main entrance to each dwelling and were considered to have an accuracy of <10 m. A database was created where each house was assigned a unique identifying code which was linked to the GPS coordinates.

### Entomologic surveys

To understand the temporal and spatial distributions of immature and adult mosquitoes throughout the village, nine surveys of approximately 100 houses (randomly selected from the list of 611 houses each survey) were undertaken over 14 months during the three seasons in central Vietnam: the dry – cool season (Jan, Mar, and Apr 2007), the dry – hot season (May and Aug 2007) and the wet – cool season (Nov 2006, Oct, Nov and Dec 2007).

Three teams of two people undertook the surveys. Permission from householders was obtained to survey their premises. Consent was given verbally from the head of the household, as this was consistent with local practice and customs for community surveys, and consent was recorded by the survey staff at the time of the survey. The type and capacity of all containers in and around the houses was recorded. For water-holding (wet) containers, the amount of water at the time of the survey was also recorded, and householders were asked about the source and use of their water. For small containers, immatures were removed using a pipette or the entire contents were emptied through a net. For larger containers, a five-sweep net sampling technique was used [18]. Wells were not sampled as they contained brackish water and were unsuitable for *Ae. aegypti* immatures. The contents of pipettes/nets were placed in 250 mL clear plastic cups. The presence/absence of I/II instar *Aedes* and *Culex* spp. and predators (e.g. *Mesocyclops* spp., *Micronecta* spp., fish spp.) was recorded. Third and IV instars and pupae were transferred into 50 mL vials for transport to the laboratory at the Institute Pasteur, Nha Trang (IPNT).

One unbaited BG-Sentinel mosquito trap (BG-trap) (BioGents AG, Regensburg, Germany) was set overnight within each house for approximately 24 h. Traps were operated from mains power and householders were financially compensated. Traps were located in the corner of a living room or bedroom (i.e. not in kitchens or bathrooms) considered to be relatively well lit, although the final location of the trap was dictated by access to electrical power (generally there is only one electrical outlet per house) and convenience to householders. When the traps were retrieved, the mesh bags containing the captured adult mosquitoes were placed in a box lined with a moist towel, and transported to the laboratory at the IPNT for identification.

Third and IV instars were identified to species under dissecting microscopes and counted together. For containers with large numbers of immatures (>50), species identifications were based on a subsample of 50 individuals. For containers with mixed populations of more than one *Aedes* species, the numbers of each species were estimated based on their proportions in the subsample. Pupae were counted and their species composition was estimated based on the identification of III/IV instars from the same container. Counts of III/IV instars and pupae from 5 sweep-net samples were then converted to estimates of total number of immatures in each container using immature stage- and container-specific correction factors [18]. Adult mosquitoes were sexed and identified to species level.

### Data analysis – temporal trends

The abundance of each life-stage (i.e. III/IV instars, pupae and adult females) was compared across survey periods with generalized linear models using a negative binomial link and Dunn-Sidak correction for multiple comparisons in SPSS. The volume of water stored per house and the number of wet containers per house was compared between survey periods with Kruskal-Wallis non-parametric tests and Dunn's pairwise multiple comparisons in SigmaStat 3.1 (Systat Software, San Jose, CA). The number of wet containers positive for III/IV instars or pupae across the survey periods was compared with contingency chi-square tests. Houses that were sampled multiple times over the study period were compared for both presence/absence of III/IV instars, pupae and females per BG-trap, and for the number of times they were considered a key premise (houses producing ≥90^th^ percentile for that life-stage/survey were defined as key premises for that life-stage/survey). Expected numbers of houses were calculated assuming all sampling events were independent and had a binomial distribution. Observed and expected numbers were compared with chi-square tests. For each life-stage/survey, the contribution of the house with the highest number of mosquitoes (and the highest 3, 5 and 10 houses) to the overall production of that life-stage, were compared across surveys using chi-square tests.

### Data analysis – spatial trends

To determine if houses having high numbers of one life-stage of *Ae. aegypti* also had high numbers of other life-stages, Spearman correlations of the abundance of III/IV instars, pupae and adult females at each house were calculated for each survey. To determine if there were areas of the village consistently producing high numbers of mosquitoes, the presence/absence of each life-stage per house, the number of *Ae. aegypti* III/IV instars and pupae per house, and the number of female *Ae. aegypti* per BG-trap, were examined over the different surveys with exploratory spatial statistics. Mean distances and degree of clustering between the 100 houses for each survey were calculated in ArcGIS 9.2 (ESRI, Redlands, USA). Spatial clusters were detected using global K-functions and the local Gi*(*d*) statistic [19] to determine any spatial structure in the weighted variables (e.g. number of pupae per house), after considering the spatial structure of the houses sampled. Briefly, K-functions (square-root transformed to L(*d*) to make the Poisson K function linear and to stabilise the variance) were first used to determine whether there was clustering of the surveyed houses beyond complete spatial randomness. Weighted K-functions (similarly transformed but to L_w_(*d*)) were then used to determine if there was clustering in the abundance (and presence/absence) of female *Ae. aegypti*, III/IV instars or pupae beyond the spatial structure of the houses. Because we were interested in determining if there were relatively large areas of the village with high or low clusters of mosquitoes, these K-function analyses were calculated for distances of 30–100 meters at 10 meter intervals. To determine if the weighted variable was more clustered than the sampled houses, we compared changes in L(*d*) and L_w_(*d*) for each 10 meter distance band [19]. Upper and lower significance boundaries were determined by a permutation procedure in which the observed values for the weighted variable being examined were resampled among the house locations 99 times [19],[20] (i.e., the 0.01 level of confidence).

Having determined the distances at which the variables of interest (i.e. abundance or presence/absence of female adults, pupae or III/IV instars per house) were displaying spatial structure independent of the structure of the sampled houses, the local Gi*(*d*) statistic was then used to identify individual houses that were members of statistically significant high or low clusters [19]. We considered a cluster significant if it contained two or more houses that either had Z scores <−2.575 or >+2.575 (the 0.01 level of confidence). For the analyses of abundance, the mean (±SD) number of III/IV instars, pupae or female mosquitoes collected from houses inside these clusters was calculated and compared to the mean (±SD) number in the remaining houses outside of these clusters. All global K-function and local Gi*(*d*) calculations were performed using the online version of Point Pattern Analysis (http://www.nku.edu/~longa/cgi-bin/cgi-tcl-examples/generic/ppa/ppa.cgi).

### Estimates of the total population of *Ae. aegypti* females in Tri Nguyen village

Estimates were made using extrapolations of our pupal survey data and assuming equilibrium conditions of constant recruitment and survival [14],[15],[21]. We used a random re-sampling with replacement method to extrapolate data from the 100 households from each survey to the 611 households throughout the village. To estimate the numbers of female *Ae. aegypti* adults emerging per house per day, we multiplied the number of pupae in each house by the daily rate of pupal development (based on water temperatures ranging from 22 to 29°C recorded in containers in Vietnam [18], and hence pupal development rates of either 0.30 or 0.49 per day, respectively [14]), the proportion of pupae that emerge as adult females (0.5) [22], and the rate of successful emergence (0.83) [22]. Based on the assumption that the mean numbers of females emerging per day was constant and the probability of daily adult survival (PDS) was constant (either 0.8 or 0.9), we calculated the total adult female population for all age classes. We then used a Monte Carlo simulation function in PopTools 3.0.3 [23] to replicate this procedure 999 times and calculate the mean number of females of all age classes and the corresponding 95% confidence limits. Separate analyses were completed for each of the nine surveys.

## Results

### Study site

**Figure 2:**
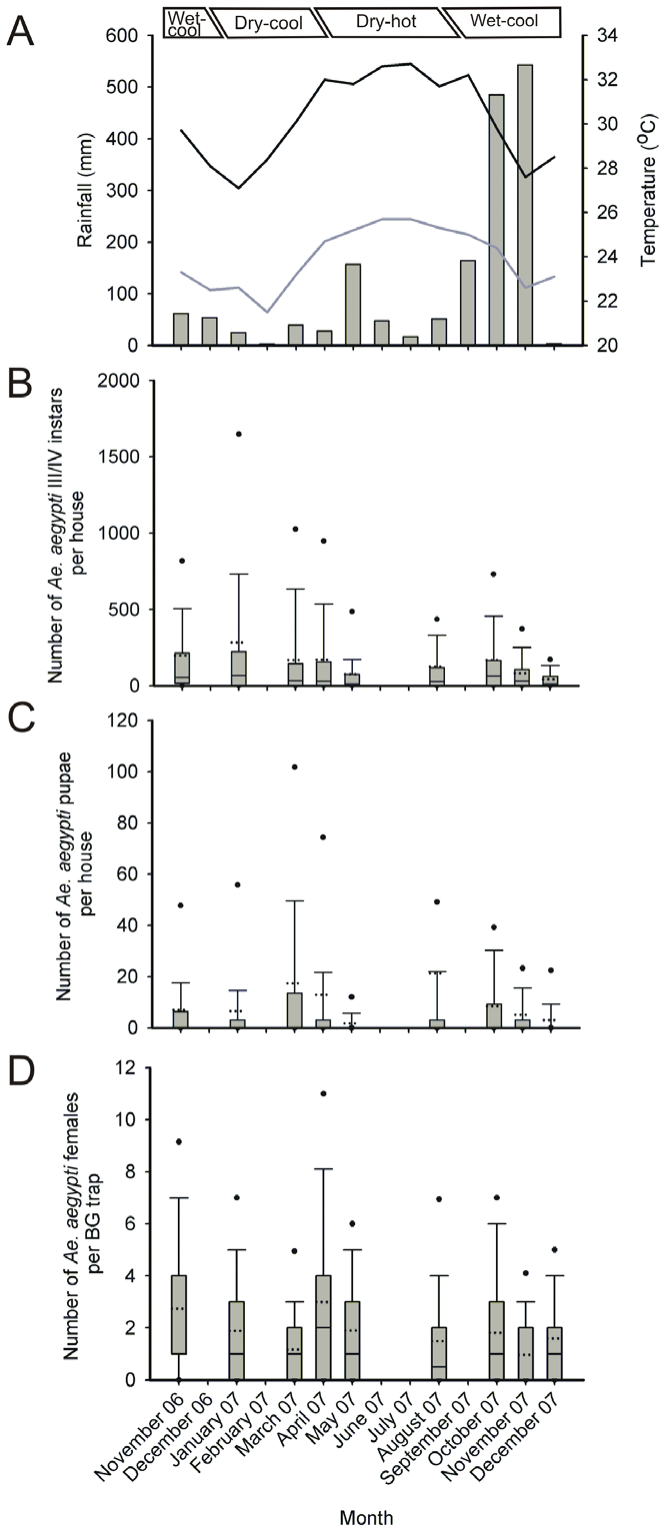
Temporal abundance of *Aedes aegypti* in Tri Nguyen village during different seasons. A) Mean monthly minimum and maximum temperatures (°C), and total monthly rainfall (mm), recorded during the survey period from Nha Trang weather station. B–D). Box plots of the number of *Ae. aegypti* III/IV instars and pupae per house, and female *Ae. aegypti* per BG-trap, for each of the surveys in Tri Nguyen village. Central line = median, dotted line = mean, box = 25^th^/75^th^ percentiles, whiskers = 10^th^/90^th^ percentiles, dots = 5^th^/95^th^ percentiles. Boxed text shows the different seasons in central Vietnam based on 5 year historical weather data.

In November 2006, there were 2377 permanent residents >15 years old and 877 persons <15 years old in Tri Nguyen village (average of 148 people/ha). Of the 611 households, 85% relied on fishing as their primary source of income, whilst 15% relied on trading or some other profession. Thirty-six percent of houses were constructed principally of concrete, 32% principally of corrugated metal, 6% a combination of concrete and metal, 20% of wood and tile construction, and 6% of various miscellaneous materials. There were 2613 artificial containers (AC) (average of 118 AC/ha and 0.8 AC/person).Total monthly rainfall, and mean monthly maximum and minimum temperatures recorded in Nha Trang can be seen in Figure 2A.

### Entomologic surveys

In all surveys, small water receptacles (e.g. discards, vases, ant traps etc) represented ≤5% of wet containers. Large water storage containers (molded tanks, cyclindrical tanks, box tanks and jars) accounted for 97% (Dec 2007) to 100% (Mar, May, Aug, Oct, Nov 2007) of the standing crop of III/IV instars, and 93% (Dec 2007) to 100% (Mar, Apr, May, Aug, Oct, Nov 2007) of the standing crop of pupae. In all surveys, *Aedes albopictus* (Skuse) III/IV instars and pupae comprised 0–2.9% and 0–3.6% of all immatures collected. The exception was in May 2007, when *Ae. albopictus* pupae comprised 12.4% of all pupae collected. However, all of these pupae came from one container. The percentage of wet containers positive for *Mesocyclops* spp. was low in every survey, ranging from 0% (Apr and May 07) to 3.8% (Mar 07). A total of 1632 female *Ae. aegypti* were collected in 888 trap-nights (there were 12 trap failures across all nine surveys). The percentage of BG-trap catches made up of female *Ae. aegypti* ranged from 40% (Oct 07) to 63% (May 07). *Aedes albopictus* never comprised more than 1% of BG-trap collections.

### Temporal trends

There were significant differences in the number of wet water storage containers per house (*H*_1,8_ = 25.5, *P*<0.01) and the mean volume of water stored per house (*H*_1,8_ = 106.3, *P*<0.01) (Table S1). There were significant differences in the number of wet containers positive/negative across surveys for III/IV instars (χ^2^_1,8_ = 76.3, *P*<0.01) and pupae (χ^2^_1,8_ = 70.9, *P*<0.01). The number of houses or BG-traps that were positive/negative for III/IV instars, pupae or females also differed significantly across surveys (χ^2^_1,8_ = 29.5, 47.8, 36.2, respectively; all *P*<0.001).

Mosquito abundance data was highly skewed (skewness and kurtosis ranged from 2.1–7.9 and 5.0–70.0 [for III/IV instars], 3.6–8.3 and 13.0–75.2 [for pupae], and 1.3–9.9 and 0.8–99.0 [for *Ae. aegypti* females]) with low median values, particularly for pupae (0 in all surveys) (Fig 2B–D). There were significant differences in the abundance of III/IV instars, pupae and females over the different survey periods (Wald χ^2^_1,8_ = 264.7, 403.7, 63.6 respectively; all *P*<0.01) (Table S1).

**Figure 3:**
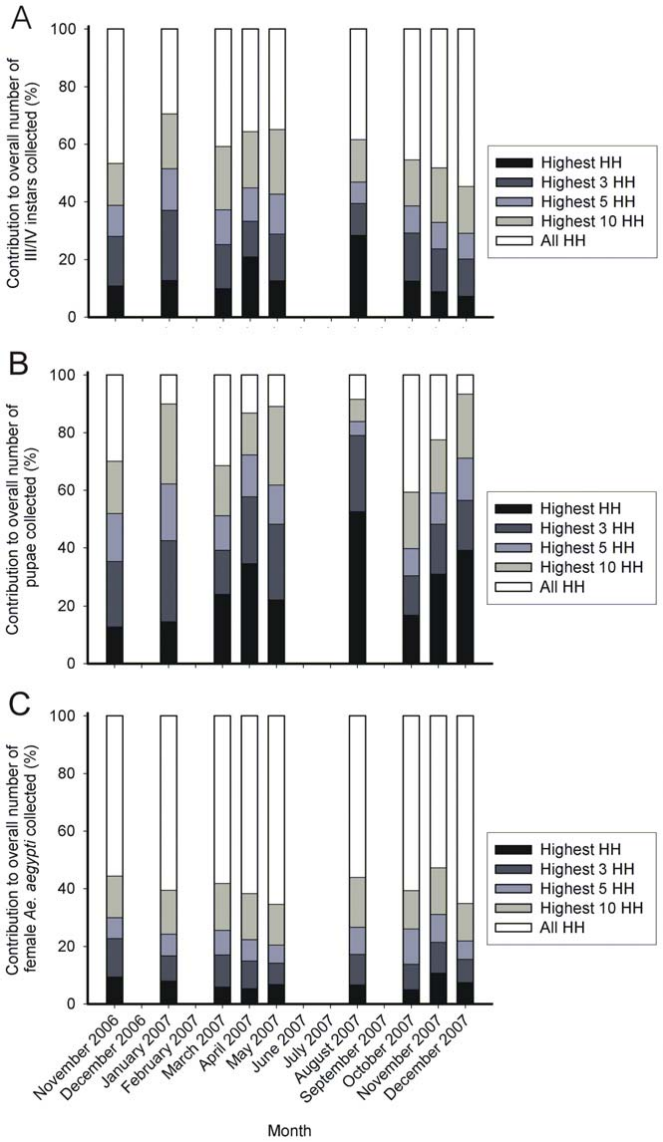
The percent contribution of the top 10, 5, 3, and highest households (HH) for each survey to the production of *Ae. aegypti*. A) III/IV instars, B) pupae and C) adult females.

Some houses contributed significantly more than others to total production of *Ae. aegypti* III/IV instars, pupae and adult females (Fig. 3). The contribution of the top, the top three, five and 10 III/IV instar producing house(s) during each survey ranged from 8–29%, 20–40%, 29–52% and 45–71% of the total population, respectively, and varied significantly between surveys (χ^2^_1,8_ = 3587.7, 1768.8, 2070.3 and 265.8, respectively; all *P*<0.001). The contribution of the top, the top three, five and 10 pupae producing house(s) during each survey ranged from 13–53%, 29–58%, 40–84% and 59–94%, respectively, and varied significantly between surveys (χ^2^_1,8_ = 774.0, 1004.5, 796.0 and 648.3, respectively; all *P*<0.001). In contrast, the contribution of the top, the top three, five and 10 BG-trap(s) to overall female *Ae. aegypti* collections during each survey was less pronounced, and ranged from 5–11%, 14–23%, 21–31% and 35–47%, respectively. These values were not significantly different between surveys (χ^2^_1,8_ = 7.1, 11.0, 10.3 and 9.6, respectively; all *P*>0.05).

### Transience of key premises

To determine whether there was any consistency in house productivity, we examined data from houses that were sampled multiple times (Tables 1 and 2). There were no significant patterns in terms of positivity of houses for III/IV instars, pupae or adult females (*P* = 0.104–0.301). Basically, houses that were positive for immatures or female adults during one survey, were no more or less likely to be positive during subsequent surveys. In terms of houses with high numbers of immatures or female adults (≥90^th^ percentile), again, there were no significant patterns in terms of their consistency across the nine surveys (*P* = 0.872–1.0). This indicated that for immature and adult female *Ae. aegypti*, key premises were transient.

**Table 1:** Number (expected number) of times households were positive for *Aedes aegypti* immatures and adult females.

Mosquito life-stage	Never positive	Positive once	Positive twice	Positive 3 times	Positive 4 times	*p-*value^a^
III/IV instars	114 (97^b^)	218 (239)	111 (117)	36 (28)	5 (4)	0.104
Pupae	269 (260)	175 (182)	31 (37)	8 (4)	1 (0)	0.260
Female *Ae. aegypti* adults	131 (121)	223 (234)	95 (102)	29 (21)	2 (2)	0.301

a Compared the observed number of times that houses scored positive for III/IV instars, pupae or females to the expected numbers.

b Expected number of sampling occasions that houses were positive for immatures or females, based on the null hypothesis that the sampling events were independent and house positivity had a binomial distribution.

**Table 2:** Number (expected number) of times households were ≥90^th^ percentile in terms of *Ae. aegypti* immature and adult female abundance.

Mosquito life-stage	Never ≥90^th^%	≥90^th^% once	≥90^th^% twice	≥90^th^% 3 times	≥90^th^% 4 times	*p*-value^a^
III/IV instars	399 (402^b^)	79 (77)	6 (5)	0 (0)	0 (0)	0.872
Pupae	402 (402)	77 (77)	5 (5)	1 (0)	0 (0)	1.000
Female *Ae. aegypti* adults	373 (372)	96 (101)	11 (9)	0 (0)	0 (0)	0.952

a Compared the observed number of times that houses had high (≥90^th^ percentile) numbers of immatures or females, compared to the expected numbers.

b Expected number of sampling occasions that houses had high (≥90^th^ percentile) numbers of immatures or females, based on the null hypothesis that the sampling events were independent and houses with high numbers of immature or females had a binomial distribution.

### Spatial trends

Spearman correlation coefficients between III/IV instars and pupae were all significant and positive, ranging from 0.36 in January 2007 to 0.70 in March 2007 (all *P*<0.01). Coefficients between III/IV instars and adult females were low and not significant, ranging from −0.02 in May 2007 to 0.18 in November 2007 (all *P*>0.05), except January 2007 (r = 0.25, *P*<0.05). Coefficients between pupae and adult females were also low and not significant, ranging from −0.05 in December 2007 to 0.13 in January 2007 (all *P*>0.05), except November 2006 (r = 0.24, *P*<0.05). These data suggest that there were no strong relationships between the abundance of immature *Ae. aegypti* at a house and the number of female mosquitoes that were collected in the BG-trap at that house.

The mean distance between houses in Tri Nguyen village is 9.1 meters. Mean distances between surveyed houses ranged from 21.3 (Dec 07) to 29.1 (Jan 07) meters. Owing to the clustering of houses in an approximate north-south axis, those houses chosen for the entomologic surveys (except Jan 07) also showed a clustered pattern at a 0.05 level of significance (Z-scores ranged from −6.64 [Dec 07] to −3.28 [Nov 06]; Z-score for Jan 07 was −1.91). This justifies the use of the K-function analyses prior to Gi*(*d*) analyses in order to account for the spatial structure of the sampled houses. For the K-function analyses, distances at which significant spatial structure (i.e. where changes in L_w_(*d*) were greater than changes in L(*d*) at a particular 10 meter distance band, and the value for L_w_(*d*) lay outside the permutation envelope) can be observed in Table S2. This showed that, globally, there was statistically significant spatial structure beyond that of the surveyed houses at various distances and amongst the different weighted variables examined.

**Figure 4:**
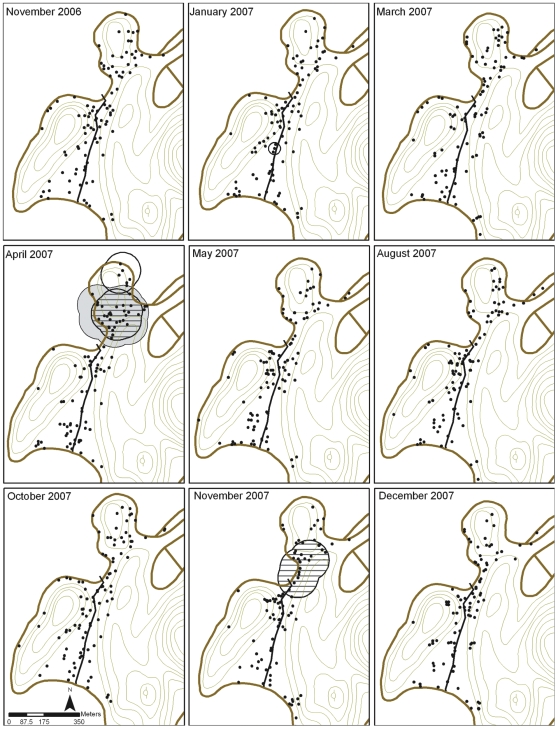
Spatial clusters of *Ae. aegypti* III/IV instar and pupae positive and negative houses in Tri Nguyen village. Horizontally hashed circles or shaded circles represent clusters of III/IV instar positive houses or pupae positive houses (*P*<0.01), respectively, after taking into consideration the structure of the houses sampled (black dots) (*P*<0.01). Clear circles represent statistically significant clusters of houses negative for III/IV instars. There was no significant spatial structure amongst houses negative for pupae, or amongst houses positive or negative for *Ae. aegypti* females (collected with BG-traps).

Local Gi*(*d*) statistics were then calculated for the weighted variables and distances deemed to be significant after K-function analyses. In terms of the presence/absence of mosquitoes in houses, there were areas in the center and northern parts of the village (Jan 07 and Apr 07, respectively) that had statistically significant clusters of houses negative for III/IV instars (Fig. 4). We also observed significant clusters of houses positive for III/IV instars (Apr and Nov 07) and pupae (Apr 07) (Fig. 4). There was no significant clustering of either negative or positive houses for *Ae. aegypti* females during any survey. These data suggest that there were no large areas of the village consistently supporting clusters of *Ae. aegypti* positive or negative houses.

**Figure 5:**
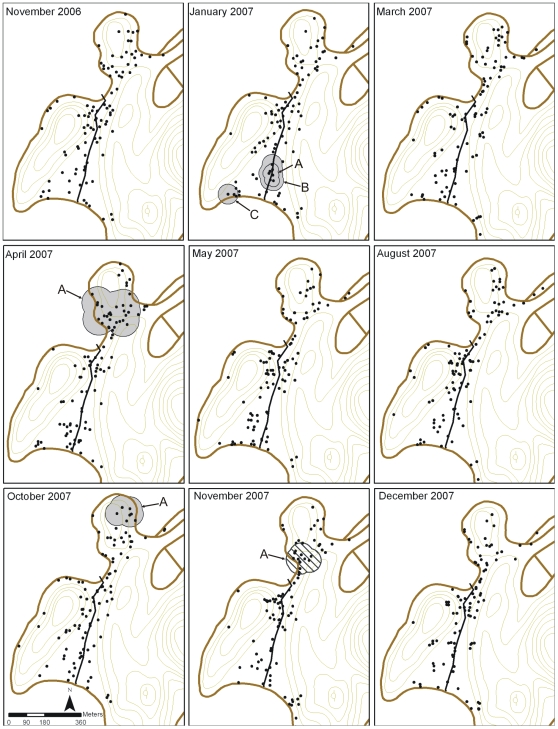
Spatial abundance of *Ae. aegypti* pupae and adult females in Tri Nguyen village. Shaded circles or diagonally-hashed circles represent statistically significant clusters of houses with high numbers of pupae (*P*<0.01), or adult females per BG-trap (*P*<0.01), respectively, after taking into consideration the structure of the houses sampled (black dots) (*P*<0.01). Letters refer to clusters whose statistics are outlined in Table 3. There was no significant spatial structure in the abundance of *Ae. aegypti* III/IV instars.

Similarly, there were areas of the village that exhibited statistically significant clustering of houses with high abundance of pupae and adult female mosquitoes, although these areas were not consistent over time (Fig. 5). For instance, significant clusters of houses producing high numbers of pupae were observed in the southern part of the village in January 2007, and in the northern part in April and October 2007. No significant clusters were detected during the other surveys. The mean (±SD) number of pupae in houses in significant clusters ranged from 32.1 (±51.6) to 34.9 (±41.0) whilst the mean number of pupae in houses not in clusters ranged from 4.3 (±13.4) to 6.7 (±12.3) (Table 3). Only one statistically significant cluster of houses with high numbers of *Ae. aegypti* females was found (Nov 2007). The mean (±SD) number of *Ae. aegypti* females in BG-traps in the houses in this cluster was 1.9 (±2.9) whilst the mean number of females in BG-traps in the houses not in the cluster was 0.8 (±1.1) (Table 3). No statistically significant groups of houses exhibited high numbers of III/IV instars during any survey.

**Table 3:** Mean number of pupae or adult female *Ae. aegypti* collected in houses that were members of the significant clusters or in houses that were not members of the significant clusters.

Survey	Cluster	Mean number±SD of pupae in houses in clusters (n)	Mean number±SD of pupae in houses not in clusters (n)	Ratio^a^	Mean number±SD of females in houses in cluster (n)	Mean number±SD of females in houses not in cluster (n)	Ratio^b^
Nov 06	—	—	—	—	—	—	—
Jan 07	A^c^	34.9±41.0 (7)	4.3±13.4 (89)	8.1	—	—	—
	B	33.7±45.3 (3)	5.7±16.7 (93)	5.9	—	—	—
	C	33.7±45.3 (3)	5.7±16.7 (93)	5.9	—	—	—
Mar 07	—	—	—	—	—	—	—
Apr 07	A	33.3±86.2 (28)	4.8±21.4 (71)	6.9	-	-	-
May 07	—	—	—	—	—	—	—
Aug 07	—	—	—	—	—	—	—
Oct 07	A	32.1±51.6 (7)	6.7±12.3 (92)	4.8	—	—	—
Nov 07	A	—	—	—	1.9±2.9 (14)	0.8±1.1 (83)	2.4
Dec 07	—	—	—	—	—	—	—

a Ratio of the mean number of pupae in houses in the cluster/mean number of pupae in houses outside the cluster.

b Ratio of the mean number of female *Ae. aegypti* in houses in the cluster/mean number of female *Ae. aegypti* in houses outside the cluster.

c Cluster letter refers to clusters found in Figure 5.

### Estimates of the standing crop of *Ae. aegypti* females

**Figure 6:**
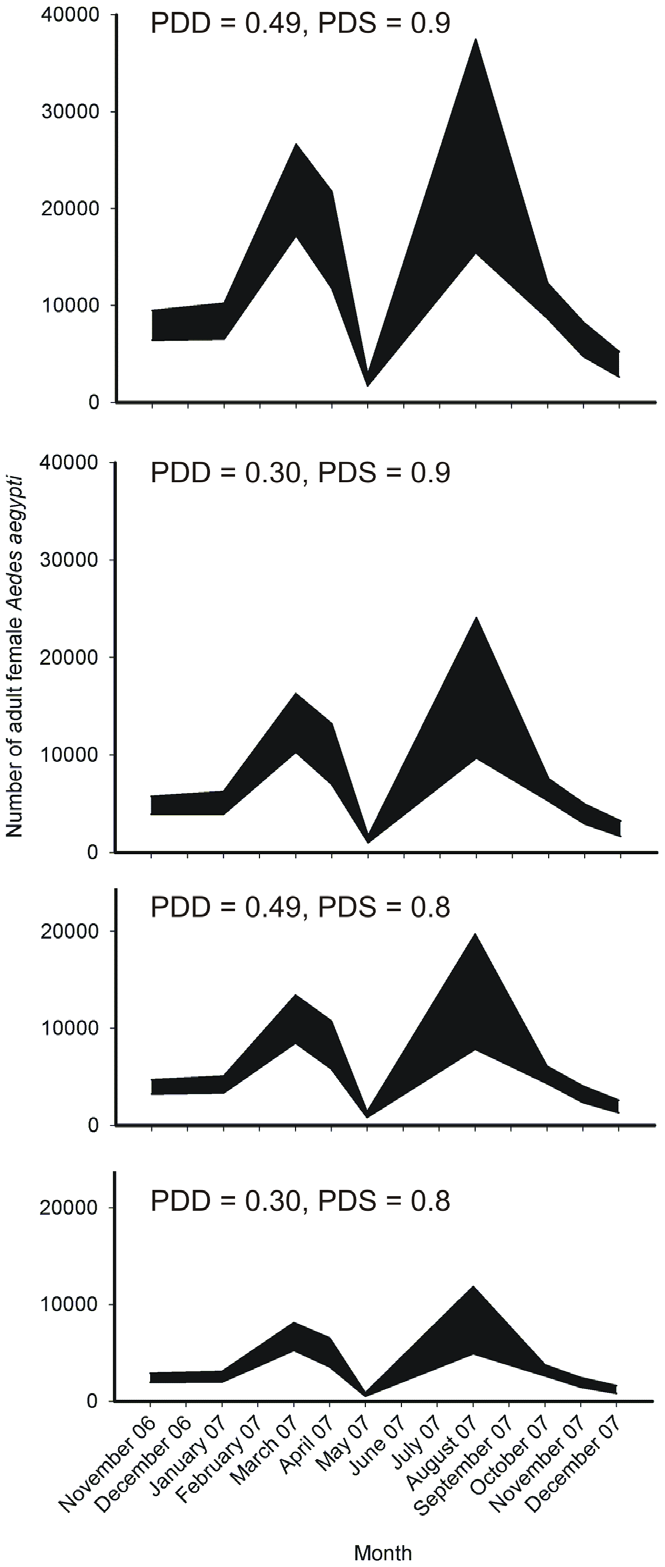
Ninety-five percent confidence limits for estimated numbers of adult female *Ae. aegypti* in Tri Nguyen village (Nov 2006 – Dec 2007). Confidence limits based on extrapolating pupal survey data from 100 households to 611 households 999 times, and assuming a pupal daily development rate (PDD) of 0.30 or 0.49, a pupal survival rate of 0.83, a female∶male sex ratio of 1∶1, and a probability of daily survival (PDS) for adults of 0.8 or 0.9.

At the faster pupal development time (0.49 d^−1^) and highest PDS (0.9), estimates of the standing crop of *Ae. aegypti* females in Tri Nguyen village ranged from 2,218 (95% CL: 1,707–2,828) when the mean number of pupae per house was 1.8 (May 07 pupal data), to 26,431 (95% CL: 15,474–37,483) when the mean number of pupae per house was 21.2 (Aug 07 pupal data) (Fig. 6). At the slower pupal development rate (0.30 d^−1^) and lowest PDS (0.8), estimates ranged from 675 (95% CL: 515–859) using May 2007 pupal data, to 8,113 (95% CL: 4,920–11,866) using August 2007 pupal data.

## Discussion

This is the first time modelling estimates of release ratios for a *Wolbachia*-based intervention have been combined with *Ae. aegypti* spatial and temporal abundance data from a potential release site to give an estimate of required release numbers. Our estimates of the potential population size, lack of seasonality and lack of consistent spatial structure, and the importance of transient key premises, will be critical for developing and guiding a pilot trial for a *Wolbachia*-based intervention.

In Tri Nguyen village, the *Ae. aegypti* adult female population size was estimated to range from 675 (95% CL: 515–859) to 26,431 (95% CL: 15,474–37,483) individuals of all age classes. A significant proportion of the 39-fold variation in these estimates is due, in part, to the inherent difficulties associated with quantitative sampling of *Ae. aegypti* pupae [18]. The highly skewed distribution of pupal counts in water containers in Tri Nguyen village means that estimates of standing crops of pupae can be strongly influenced by a few containers with extremely high counts. Because we sampled approximately 16% of all the households in the village each survey, premises producing high numbers of *Ae. aegypti* may have been missed, influencing our population estimates and spatial cluster detection. This might explain our low population estimates based on May 2007 data. Nevertheless, these estimates are likely to be broadly indicative of the adult female population sizes and spatial structure present in the village.

Generally, our population estimates are consistent with population estimates from other areas. For example, the *Ae. aegypti* pupal densities in Tri Nguyen village, calculated on a per person basis (range 0.34–3.98), were similar to those observed in Honduras (0.34) [24] and Iquitos (0.34–1.23 [25] and 0.21–0.53 [16]), but considerably less than those observed in Trinidad (range 1.4–63.4, mean 22.7) [26]. Our estimates of female *Ae. aegypti* population size were also similar to those calculated in Thailand (5–50 adult females per house) assuming 0.9 PDS and a 0.5 d^−1^ PDD [15]. Our estimates, if averaged per house, give a range of approximately 3.6–43.3 females per house (at 0.9 PDS and 0.49 d^−1^ PDD). The highly aggregated distribution of *Ae. aegypti* pupae that we observed has also been noted in Thailand [27], New Orleans, USA [13], and Australia [28].

Although there were significant differences in immature and adult female mosquito prevalence and abundance across the nine surveys, these differences did not appear to be related to temperature and/or rainfall. Despite the increased number of wet containers per house in November 2007, the increased volume of water stored per house in November and December 2007, and the large amount of rainfall in October and November 2007, this did not translate into an increase in the number of III/IV instar or pupae positive containers during the same months or an increase in the proportion of positive premises for III/IV instars and pupae, or positive BG-traps for female *Ae. aegypti*. This is most likely due to the peridomestic nature of *Ae. aegypti* immature habitats, and the fact that the availability of wet containers is not always driven by rainfall [2], particularly in Tri Nguyen village, where discards/small jars are fewer and less important compared to larger and more permanent water storage containers, and where water is sometimes purchased from vendors. However, it may also reflect the fact that our study spanned just over one year – detection of consistent seasonal patterns of mosquito abundance would require longer sampling periods. For instance, seasonal variation in *Ae. aegypti* abundance was observed in Iquitos, Peru over a 3.5 year sampling period [16]. In Thailand, *Ae. aegypti* abundance was associated with average temperatures 6 wk prior, although no association with rainfall was observed (3 year sampling period) [29]. In Puerto Rico, no association between rainfall or temperature and *Ae. aegypti* abundance (2 year sampling period) was found, although only temperature showed a distinct seasonal pattern [29].

Spatially, at a village-wide level, there were no areas of Tri Nguyen village that consistently supported high numbers of mosquitoes, or clusters of houses consistently positive or negative for mosquitoes. This is similar to results from Thailand, in which areas of concentrated *Ae. aegypti* abundance varied seasonally [15]. This contrasts with results found in Iquitos, Peru, in which the Maynas zone had consistently high levels of *Ae. aegypti* infestation [16], although the scale of their study (∼6000 houses over ∼16 km^2^) was larger than ours. At the house level, the mosquito population in Tri Nguyen village at any one time point was heterogeneous and varied considerably within relatively short periods of time, a situation that was also observed in Iquitos [19], and which led those authors to conclude that the appropriate scale for assessing mosquito density in Iquitos is the household level at frequent time intervals [16],[19].

We also observed that adult females were distributed differently to larvae and pupae, with key premises for female *Ae. aegypti* contributing less to overall female abundance compared to the contribution of key premises for immatures and their contribution to overall immature production. There was also less spatial structure detected in female *Ae. aegypti* abundance, most likely due to adult dispersal. This could assist the spread of *Wolbachia* from house to house when infected females are released, particularly considering the close proximity of houses in Tri Nguyen village.

It has been proposed that estimates of the average standing crop of pupae for different container types could be extrapolated to larger areas so that risk assessments and control strategies could be streamlined to include only counts of containers by type and number of humans [30]. Similarly, it has been suggested that dengue vector control programs in Thailand could increase their efficiency by concentrating efforts primarily on areas with the greatest abundance of larvae [15]. However, the problem with this targeted approach is that the distribution of *Ae. aegypti* infested containers and households can be highly clustered through time and space within communities, making results from one-time small-scale surveys sensitive to sampling error and variation [16]. Hence, longitudinal field studies are critical [29]. We have demonstrated that there is little consistency in the location of key premises in Tri Nguyen village, particularly in terms of the absolute abundance of *Ae. aegypti* immatures or adults. In the context of *Ae. aegypti* control in Vietnam, programs that specifically target key premises are unlikely to succeed, unless a practical method that can predict or rapidly identify key premises immediately prior to an intervention is developed. In contrast to our data, considerable stability was observed in key premises in north Queensland [28] and Trinidad [31], respectively. In north Queensland, positive premises were three times more likely to remain positive over time than a negative premise was to become positive over the course of a year [28]. However, the comparison only involved two surveys over two years, while the work in Trinidad only compared three consecutive monthly surveys in one wet season [31]. One of our key findings was that, for pupae and female *Ae. aegypti* at least, although the proportion of positive households varied significantly between different times of year, overall production from positive houses remained relatively constant, with most variation driven by a few key premises. However, the location of both positive and key premises is transient. Thus our study highlights the importance of multiple surveys, particularly when trying to evaluate the spatial structure of a vector population – if any of our surveys are considered in isolation from the others, conclusions regarding key premises or areas of the village that support significantly high numbers of mosquitoes may be misleading.

For an intervention involving the release of *w*MelPop transinfected *Ae. aegypti*, the number of insects to be released, the frequency of release and area to be covered need to be known. Because adult dispersal is greater than that of immatures, adult releases are more likely to be successful when covering a large area [11],[12] and it is logical to release males as well as females because this avoids the issue of sexing before release. The unstable equilibrium [6] or the *Wolbachia* introduction threshold, which is the frequency of infected individuals in the population that must be exceeded for *Wolbachia* to successfully be established [9], has been estimated for mosquito populations to be approximately 15–45% (release ratios of 0.18∶1–0.82∶1). This depends on the level of delayed mortality experienced by the *Wolbachia-*infected mosquitoes [9],[11] and any deleterious fitness effects due to the *Wolbachia* infection apart from longevity effects. Assuming that the delayed mortality observed in *w*MelPop transinfected *Ae. aegypti* in the laboratory [10] will also be observed in the field, then the introduction threshold is likely to be around 40% or a release ratio of 0.67∶1 (M. Turelli, personal communication). However, these calculations were based on the assumptions of no population age-structure and discrete, non-overlapping generations, something which is unlikely to occur with *Ae. aegypti* field populations. For *Ae. aegypti* populations, a field release of all age-classes in sufficient numbers to exceed the introduction threshold in each age class would be required, or the release of sufficient numbers of a sub-set of age classes so that frequency of infection in their offspring and subsequent generations will eventually exceed the unstable equilibrium. A field release of all age-classes would be logistically harder than a release of a subset of age classes. Furthermore, models have indicated that the release of infected gravid adults will result in more rapid invasion and lower required introduction thresholds compared to releases of teneral adults, although releasing gravid females is technically harder than releasing teneral adults [11]. For a release of teneral *w*MelPop transinfected adults (females and males) into an age-structured population of uninfected *Ae. aegypti*, Rasgon and Scott [11] estimated that the release ratios would need to be 5- to 10-fold higher than estimates from non age-structured models [6]. For gravid mosquitoes, release ratios into age-structured populations only need to be 1.2- to 2.75-fold higher than that into non age-structured populations.

Whatever the strategy chosen, it is critical for a release to exceed the unstable equilibrium. We found that *Ae. aegypti* adult female populations in Tri Nguyen could range from 675 (95% CL 515–859) to 26,431 individuals (95% CL 15,474–37,483), with little association observed between season and abundance. Based on the Rasgon and Scott [11] and M. Turelli (personal communication) estimates above, we calculate that release ratios of teneral adults (*Wolbachia* infected females and males) to uninfected adults of all classes would need to exceed 3.4–6.7∶1. Consequently, if a release occurred during a time of maximum mosquito abundance, this could equate to releasing up to 89,865–177,088 (95% CL 52,612–251,136) adult females and males, or an average of 147–290 (95% CL 86–411) females and males per house. If gravid females and similarly aged males were to be released, then the ratios of infected to uninfected adults would be less (0.8–1.8∶1), and similarly the numbers of mosquitoes to release per house would be less (34–78 females and males, 95% CL 20–110).

Relative to the actual number of mosquitoes per house that we observed in Tri Nguyen village, our estimates for a release are high, and raise logistical and ethical questions, particularly regarding community engagement and authorization [32]. To counter this, we consider that pre-release interventions to reduce adult numbers will be required [11]. In Tri Nguyen village, in the absence of a larvicide registered for use against *Ae. aegypti* in Vietnam, it would seem feasible to implement control by a simple netting procedure in large containers which produce the bulk of the *Ae. aegypti* population. This could significantly reduce the release numbers, effectively meaning that such releases would not result in a net increase in adult mosquito numbers. This would help to ensure that any proposed final release strategy will also be amenable to community engagement and authorization [32]. Based on our findings of the spatial distribution of *Ae. aegypti* in Tri Nguyen, our future models need to consider the feasibility and effect of conducting multiple releases over a period of several days/weeks, thus further reducing required release numbers at any given time [11]. Multiple releases will also likely help to counteract any short term temporal heterogeneities in wild mosquito numbers and dampen the effect of transient key premises. Considering our population estimates and the relative ease of rearing *Ae. aegypti* in the laboratory, release of sufficient numbers throughout the village to achieve a stable equilibrium would be feasible.

The next step in the development of a pilot release of *Wolbachia* transinfected *Ae. aegypti* in Tri Nguyen village will be to develop spatially explicit models that can measure the impact of different release strategies on the spread of *Wolbachia,* including the time for infection to reach fixation. Ideally, these models will take into account *Ae. aegypti* dispersal patterns [17] and the wave-like nature of *Wolbachia* spread in populations [6], and also include field-derived estimates for adult mosquito survivorship. The latter is particularly important if we are to calculate the potential impacts of insect life-shortening strategies on pathogen transmission risk.

## Supporting Information

Table S1 Summary of results from surveys for *Aedes aegypti* in Tri Nguyen village, Vietnam.
(0.03 MB DOC)

Table S2 Results from the K-function analyses for *Aedes aegypti* presence/absence and abundance. These were calculated at distances of 30 - 100 meters, at 10 meter intervals, and show the distances at which the weighted variable was clustered more than that of the surveyed houses.
(0.04 MB DOC)
