# Supplementary material for: Characterizing the Aedes aegypti Population in a Vietnamese Village in Preparation for a Wolbachia-Based Mosquito Control Strategy to Eliminate Dengue
Source: PLoS Negl Trop Dis. 2009 Nov 24;3(11):e552. doi: 10.1371/journal.pntd.0000552 (PMC2780318; doi:10.1371/journal.pntd.0000552)
Supplement: Table S1 — Summary of results from surveys for Aedes aegypti in Tri Nguyen village, Vietnam. (0.03 MB DOC) [file pntd.0000552.s001.doc]

**Table S1.** Summary of results from surveys for *Aedes aegypti* in Tri Nguyen village, Vietnam.

| **Survey** | **No. of wet containers surveyed per house** | **Approximate volume of water stored per house (liters)** | **% of houses +ve for III/IV instars** | **No. of containers +ve for III/IV per house** | **% of houses +ve for pupae** | **No. of containers +ve for pupae per house** | **No. of III/IV instars per house** | **No. of pupae per house** | **% of BG-traps +ve for females** | **No. of females per BG-trap** |
| --- | --- | --- | --- | --- | --- | --- | --- | --- | --- | --- |
| Nov 06 | 3.5 ab^1^ | 2130 cd | 81 | 1.7 | 41 | 0.6 | 197.0 a | 7.0 a | 76 | 2.7 a |
| Jan 07 | 3.0 a | 1650 d | 75 | 1.4 | 30 | 0.4 | 284.8 a | 6.6 a | 56 | 1.9 ab |
| Mar 07 | 3.2 a | 1490 d | 68 | 1.2 | 48 | 0.7 | 168.5 a | 17.3 b | 51 | 1.2 bc |
| Apr 07 | 3.7 ab | 2120 bcd | 63 | 1.1 | 29 | 0.4 | 170.7 a | 12.9 bc | 73 | 3.0 a |
| May 07 | 3.3 ab | 2290 bc | 59 | 1.0 | 18 | 0.2 | 75.1 b | 1.8 e | 62 | 1.9 ac |
| Aug 07 | 3.4 ab | 1780 cd | 56 | 0.9 | 32 | 0.4 | 126.6 ab | 21.2 b | 50 | 1.5 ac |
| Oct 07 | 3.2 a | 1850 bcd | 72 | 1.3 | 41 | 0.6 | 169.1 a | 8.5 a | 55 | 1.8 ac |
| Nov 07 | 4.2 b | 3560 a | 61 | 1.1 | 26 | 0.3 | 83.1 b | 5.1 af | 44 | 1.0 bc |
| Dec 07 | 3.4 ab | 2780 abc | 54 | 1.0 | 13 | 0.2 | 43.0 c | 3.1 ef | 61 | 1.6 ac |

^1^Values followed by a different letter were significantly different at *P* < 0.01.
