# Supplementary material for: Characterizing the Aedes aegypti Population in a Vietnamese Village in Preparation for a Wolbachia-Based Mosquito Control Strategy to Eliminate Dengue
Source: PLoS Negl Trop Dis. 2009 Nov 24;3(11):e552. doi: 10.1371/journal.pntd.0000552 (PMC2780318; doi:10.1371/journal.pntd.0000552)
Supplement: Table S2 — Results from the K-function analyses for Aedes aegypti presence/absence and abundance. These were calculated at distances of 30 - 100 meters, at 10 meter intervals, and show the distances at which the weighted variable was clustered more than that of the surveyed houses. (0.04 MB DOC) [file pntd.0000552.s002.doc]

**Table S2**. Results from the K-function analyses for *Aedes aegypti* presence/absence and abundance. These were calculated at distances of 30 – 100 meters, at 10 meter intervals, and show the distances at which the weighted variable was clustered more than that of the surveyed houses.

| **Survey** | **Weighted variable** | **III/IV instars** | **Pupae** | **Adult females** |
| --- | --- | --- | --- | --- |
| November 2006 | Positive/negative | 30, 40, 70, 80, 90, 100 | 30, 40, 70, 80, 90, 100 | 30, 100 |
|  | Abundance | 50, 60 | 100 | 40, 80 |
| January 2007 | Positive/negative | **30^1^**, 50, 80 | 30 | 50, 70, 80, 100 |
|  | Abundance | 40 | **30**, **50**, 100 | 80 |
| March 2007 | Positive/negative | 40, 50, 90, 100 | 40, 60, 70, 80, 100 | - |
|  | Abundance | 20, 50, 90 | 50, 100 | 50, 90 |
| April 207 | Positive/negative | 30, **50**, **70**, **80**, **90** | 30, 50, 60, **70**, **80**, **90**, **100** | 30, **40**, **50**, **60**, **70**, 100 |
|  | Abundance | 60, 70, 80, 90 | 40, 70, **80** | 40, 80, 90 |
| May 2007 | Positive/negative | **100** | 40, 100 | **30**, **40**, **60** |
|  | Abundance | - | 70, 100 | 30, 80, 100 |
| August 2007 | Positive/negative | 40, 50 | 60, 80, 90 | 30, 70 |
|  | Abundance | 40, 50 | - | 30 |
| October 2007 | Positive/negative | 70 | 30, 80 | 30, 80, 100 |
|  | Abundance | 100 | 50, **60** | 40, 80 |
| November 2007 | Positive/negative | 40, 60, 70, **80**, **90**, **100** | 40, 70 | 50, 60 |
|  | Abundance | 70, 80, 90 | 70 | 40, 50, **60** |
| December 2007 | Positive/negative | 50, 80 | - | 30, 40, 70 |
|  | Abundance | 80 | - | 30, 60, 70, 80, 100 |

^1^Bold values represent those distances at which the value of the weighted variable was significant at *P* < 0.01.
